# Congenital cytomegalovirus screening by dried blood spot: a systematic review

**DOI:** 10.1007/s00431-026-07196-8

**Published:** 2026-06-25

**Authors:** Raquel Amaral Machado, Isadora Soares Bicalho Garcia, Guilherme Soares de Albuquerque, Juliana Luiza Paula de Araújo, Rafaela Coelho Gonçalves, Noélida Mendes Rodrigues de Almeida, Rafael dos Santos Borges, Ericka Viana Machado Carellos, Roberta Maia de Castro Romanelli

**Affiliations:** 1 Universidade Federal de Minas Gerais, Belo Horizonte, Minas Gerais Brazil; 2 Department of Pediatrics, Universidade Federal de Minas Gerais, Belo Horizonte, Minas Gerais Brazil

**Keywords:** Congenital, Cytomegalovirus, Dried blood spot, Screening

## Abstract

Congenital human cytomegalovirus (cCMV) infection is a leading cause of sensorineural hearing loss and neurological sequelae in children. Although newborn screening strategies remain controversial, dried blood spots (DBS) collected for routine metabolic screening have been proposed as a low-cost method for large-scale detection. This systematic review assessed studies that evaluated the accuracy of DBS testing for screening newborns for cCMV infection. A systematic review was conducted following the *Cochrane Handbook for Systematic Reviews of Diagnostic Test Accuracy* and reported in accordance with PRISMA-DTA. The PIRT strategy included the following: Population (newborns undergoing screening for cCMV), Index test (detection of cCMV using DBS samples), Reference standard (confirmatory testing using saliva or urine PCR collected within 21 days of life), Target condition (cCMV infection). Searches were performed in PubMed/MEDLINE, Scopus, Bireme, Embase, LILACS, SciELO, and CENTRAL. The search identified 365 articles; after removing 210 duplicates, 155 were screened by title and abstract, and 9 studies were included. The included studies tested between 1174 and 551,034 DBS samples each. Confirmatory testing used PCR in urine (seven studies) or saliva (two studies). Three studies used paired samples for screening; sensitivity ranged from 28.3% to 79.3%, specificity from 99.9% to 100%. Positive predictive value (PPV) ranged from 39 to 100%, and negative predictive value (NPV) from 99.6% to 99.9%. *Conclusion*: Neonatal screening for congenital cytomegalovirus using DBS samples is promising, since it would allow early identification for timely interventions to reduce sequelae.

**Table:** 

**What is Known:** *• Congenital CMV is a leading cause of sensorineural hearing loss and neurodevelopmental sequelae, with many cases asymptomatic at birth. Diagnosis relies on early viral detection, and while saliva/urine PCR is sensitive, newborn screening lacks consensus and feasibility is unknown in many settings.*	
**What is New:** *• DBS PCR emerges as a scalable, feasible low-cost screening alternative for cCMV screening. A few states in the USA and provinces in Canada have implemented newborn cCMV screening programs using DBS, but the sensitivity appears lower than in research studies. Improving DNA extraction and PCR methods are required as well as assessing feasibility of using other specimens (e.g., urine or saliva) for screening.*

**What is Known:**

*• Congenital CMV is a leading cause of sensorineural hearing loss and neurodevelopmental sequelae, with many cases asymptomatic at birth. Diagnosis relies on early viral detection, and while saliva/urine PCR is sensitive, newborn screening lacks consensus and feasibility is unknown in many settings.*

**What is New:**

*• DBS PCR emerges as a scalable, feasible low-cost screening alternative for cCMV screening. A few states in the USA and provinces in Canada have implemented newborn cCMV screening programs using DBS, but the sensitivity appears lower than in research studies. Improving DNA extraction and PCR methods are required as well as assessing feasibility of using other specimens (e.g., urine or saliva) for screening.*

## Introduction

Congenital human cytomegalovirus infection is a leading cause of sensorineural hearing loss and other neurological sequelae in children. In developed countries, such as the USA, Canada, and Western Europe, its prevalence is approximately 0.85%, whereas in African regions, it is significantly higher, reaching about 3.71% [1]. In Brazil, screening for cCMV in newborns lacks specific guidelines due to cost-effectiveness concerns and the absence of consensus in screening strategies, a scenario also observed in other countries [2].

Approximately 1 in 8 neonates with cCMV presents clinical signs at birth. Among those asymptomatic at birth, some may later develop sensorineural hearing loss and other neurological complications during early childhood. Therefore, newborn screening for cCMV infection may provide an opportunity for early identification of infected neonates that should be monitored for early detection and intervention for hearing loss and neurodevelopmental delays [4]. Neonates symptomatic at birth have a higher risk of central nervous system involvement, with 40%–60% developing permanent neurological sequelae, including sensorineural hearing loss (SNHL), cognitive delay, epilepsy, visual impairment, and cerebral palsy [5]. In contrast, approximately 13.5% of those asymptomatic at birth develop long-term sequelae, most commonly SNHL [6].

For many years, tissue culture was the gold standard for cytomegalovirus (CMV) diagnosis, using cell lines, particularly human fibroblasts, that show a characteristic cytopathic effect. However, this method has been largely replaced by molecular techniques. Diagnosis of cCMV requires viral isolation or detection of viral DNA, mainly in urine or saliva, which contain high viral loads, and samples must be collected within the first 3 weeks of life to ensure accuracy [2].

PCR testing using DBS samples has been investigated as a feasible, low-cost, high-throughput option for newborn cCMV screening [7–9]. However, its sensitivity is lower than saliva-based PCR. Recent studies suggest that improved DNA extraction and PCR methods can increase DBS sensitivity, although analytical limitations persist [7].

Current knowledge gaps include the need for population-based studies to evaluate the clinical sensitivity of improved DBS screening methods and to determine the prognostic value of viral load across different sample types [2, 3].

This review assessed studies that evaluated accuracy of DBS testing for screening newborns for cCMV infection.

## Materials and methods

This systematic review followed the *Cochrane Handbook for Systematic Reviews of Diagnostic Test Accuracy* and was reported in accordance with the PRISMA-DTA guidelines [10]. The review protocol was registered in PROSPERO CRD42025630946. The research question was as follows: “In newborns, how accurate is testing of DBS samples for the detection of cCMV infection compared with confirmatory testing using of saliva or urine PCR?”.

The research question was structured according to the PIRT framework, comprising:

- Population: Neonates undergoing screening for congenital cytomegalovirus
- Index test: Testing of DBS samples for cCMV detection
- Reference standard for diagnosis: Confirmatory testing using saliva or urine PCR
- Target condition: Congenital cytomegalovirus infection

To identify diagnostic accuracy studies evaluating DBS testing for cCMV in newborns, systematic searches were conducted in PubMed (via MEDLINE), Scopus, Bireme, Embase, LILACS, SciELO, and the Cochrane Central Register of Controlled Trials (CENTRAL) (via the Cochrane Library). No restrictions were applied on language, publication date, document type, publication status, or geographic location.

The search terms used were as follows: “Neonatal Screening”; “Neonatal Screenings”; “Screening, Neonatal”; “Screenings, Neonatal”; “Newborn Screening”; “Newborn Screenings”; “Screening, Newborn”; “Screenings, Newborn”; “Infant, Newborn, Screening”; “Newborn Infant Screening”; “Newborn Infant Screenings”; “Screening, Newborn Infant”; “Screenings, Newborn Infant”; “Congenital Cytomegalovirus”; “Congenital Cytomegalovirus Infection”; “Congenital Cytomegalovirus Infections”; “Cytomegalovirus Infection, Congenital”; “Infection, Congenital Cytomegalovirus”; “Congenital CMV Infection”; “CMV Infection, Congenital”; “Congenital CMV Infections”; “Infection, Congenital CMV”; “cCMV”; “Dried Blood Spot”; “Dried Blood Spots”; “Dried Blood Spot Method”; “Dried Blood Spot Analysis”; “DBS”; “Guthrie card.” The search strategy was developed using MeSH and DeCS descriptors and adapted for each database and detailed in Table 1.

**Table 1:** Search strategies by database for the systematic review

Database	Search Strategy
SCOPUS	TITLE-ABS-KEY (("Neonatal Screening" OR "Neonatal Screenings" OR "Screening, Neonatal" OR "Screenings, Neonatal" OR "Newborn Screening" OR "Newborn Screenings" OR "Screening, Newborn" OR "Screenings, Newborn" OR "Infant, Newborn, Screening" OR "Newborn Infant Screening" OR "Newborn Infant Screenings" OR "Screening, Newborn Infant" OR "Screenings, Newborn Infant") AND ("Congenital Cytomegalovirus" OR "Congenital Cytomegalovirus Infection" OR "Congenital Cytomegalovirus Infections" OR "Cytomegalovirus Infection, Congenital" OR "Infection, Congenital Cytomegalovirus" OR "Congenital CMV Infection" OR "CMV Infection, Congenital" OR "Congenital CMV Infections" OR "Infection, Congenital CMV" OR "cCMV") AND ("Dried Blood Spot" OR "Dried Blood Spots" OR "Dried Blood Spot Method" OR "Dried Blood Spot Analysis" OR "DBS" OR "Guthrie card"))
Embase	('neonatal screening'/exp OR 'neonatal screening' OR 'neonatal screenings' OR 'screening, neonatal' OR 'screenings, neonatal' OR 'newborn screening'/exp OR 'newborn screening' OR 'newborn screenings' OR 'screening, newborn'/exp OR 'screening, newborn' OR 'screenings, newborn' OR 'infant, newborn, screening' OR 'newborn infant screening' OR 'newborn infant screenings' OR 'screening, newborn infant' OR 'screenings, newborn infant') AND ('congenital cytomegalovirus' OR 'congenital cytomegalovirus infection'/exp OR 'congenital cytomegalovirus infection' OR 'congenital cytomegalovirus infections' OR 'cytomegalovirus infection, congenital' OR 'infection, congenital cytomegalovirus' OR 'congenital cmv infection'/exp OR 'congenital cmv infection' OR 'cmv infection, congenital' OR 'congenital cmv infections' OR 'infection, congenital cmv' OR 'ccmv') AND ('dried blood spot'/exp OR 'dried blood spot' OR 'dried blood spots'/exp OR 'dried blood spots' OR 'dried blood spot method' OR 'dried blood spot analysis' OR 'dbs' OR 'guthrie card'/exp OR 'guthrie card')
PubMed	("Neonatal Screening” OR “Neonatal Screenings” OR “Screening, Neonatal” OR “Screenings, Neonatal” OR “Newborn Screening” OR “Newborn Screenings” OR “Screening, Newborn” OR “Screenings, Newborn” OR “Infant, Newborn, Screening” OR “Newborn Infant Screening” OR “Newborn Infant Screenings” OR “Screening, Newborn Infant” OR “Screenings, Newborn Infant”) AND (“Congenital Cytomegalovirus” OR “Congenital Cytomegalovirus Infection” OR “Congenital Cytomegalovirus Infections” OR “Cytomegalovirus Infection, Congenital” OR “Infection, Congenital Cytomegalovirus” OR “Congenital CMV Infection” OR “CMV Infection, Congenital” OR “Congenital CMV Infections” OR “Infection, Congenital CMV” OR “cCMV”) AND (“Dried Blood Spot” OR “Dried Blood Spots” OR “Dried Blood Spot Method” OR “Dried Blood Spot Analysis” OR “DBS” OR “Guthrie card”)
BIREME	("Neonatal Screening" OR "Neonatal Screenings" OR "Screening, Neonatal" OR "Screenings, Neonatal" OR "Newborn Screening" OR "Newborn Screenings" OR "Screening, Newborn" OR "Screenings, Newborn" OR "Infant, Newborn, Screening" OR "Newborn Infant Screening" OR "Newborn Infant Screenings" OR "Screening, Newborn Infant" OR "Screenings, Newborn Infant") AND ("Congenital Cytomegalovirus" OR "Congenital Cytomegalovirus Infection" OR "Congenital Cytomegalovirus Infections" OR "Cytomegalovirus Infection, Congenital" OR "Infection, Congenital Cytomegalovirus" OR "Congenital CMV Infection" OR "CMV Infection, Congenital" OR "Congenital CMV Infections" OR "Infection, Congenital CMV" OR "cCMV") AND ("Dried Blood Spot" OR "Dried Blood Spots" OR "Dried Blood Spot Method" OR "Dried Blood Spot Analysis" OR "DBS" OR "Guthrie card")
LILACS	("Neonatal Screening" OR "Neonatal Screenings" OR "Screening, Neonatal" OR "Screenings, Neonatal" OR "Newborn Screening" OR "Newborn Screenings" OR "Screening, Newborn" OR "Screenings, Newborn" OR "Infant, Newborn, Screening" OR "Newborn Infant Screening" OR "Newborn Infant Screenings" OR "Screening, Newborn Infant" OR "Screenings, Newborn Infant") AND ("Congenital Cytomegalovirus" OR "Congenital Cytomegalovirus Infection" OR "Congenital Cytomegalovirus Infections" OR "Cytomegalovirus Infection, Congenital" OR "Infection, Congenital Cytomegalovirus" OR "Congenital CMV Infection" OR "CMV Infection, Congenital" OR "Congenital CMV Infections" OR "Infection, Congenital CMV" OR "cCMV") AND ("Dried Blood Spot" OR "Dried Blood Spots" OR "Dried Blood Spot Method" OR "Dried Blood Spot Analysis" OR "DBS" OR "Guthrie card")
Cochrane Library	("Neonatal Screening" OR "Neonatal Screenings" OR "Screening, Neonatal" OR "Screenings, Neonatal" OR "Newborn Screening" OR "Newborn Screenings" OR "Screening, Newborn" OR "Screenings, Newborn" OR "Infant, Newborn, Screening" OR "Newborn Infant Screening" OR "Newborn Infant Screenings" OR "Screening, Newborn Infant" OR "Screenings, Newborn Infant") AND ("Congenital Cytomegalovirus" OR "Congenital Cytomegalovirus Infection" OR "Congenital Cytomegalovirus Infections" OR "Cytomegalovirus Infection, Congenital" OR "Infection, Congenital Cytomegalovirus" OR "Congenital CMV Infection" OR "CMV Infection, Congenital" OR "Congenital CMV Infections" OR "Infection, Congenital CMV" OR "cCMV") AND ("Dried Blood Spot" OR "Dried Blood Spots" OR "Dried Blood Spot Method" OR "Dried Blood Spot Analysis" OR "DBS" OR "Guthrie card")
SciELO	("Neonatal Screening" OR "Neonatal Screenings" OR "Screening, Neonatal" OR "Screenings, Neonatal" OR "Newborn Screening" OR "Newborn Screenings" OR "Screening, Newborn" OR "Screenings, Newborn" OR "Infant, Newborn, Screening" OR "Newborn Infant Screening" OR "Newborn Infant Screenings" OR "Screening, Newborn Infant" OR "Screenings, Newborn Infant") AND ("Congenital Cytomegalovirus" OR "Congenital Cytomegalovirus Infection" OR "Congenital Cytomegalovirus Infections" OR "Cytomegalovirus Infection, Congenital" OR "Infection, Congenital Cytomegalovirus" OR "Congenital CMV Infection" OR "CMV Infection, Congenital" OR "Congenital CMV Infections" OR "Infection, Congenital CMV" OR "cCMV") AND ("Dried Blood Spot" OR "Dried Blood Spots" OR "Dried Blood Spot Method" OR "Dried Blood Spot Analysis" OR "DBS" OR "Guthrie card")

Publication selection involved initial screening of titles and abstracts to assess relevance and inclusion criteria, followed by full-text review when necessary. Inclusion criteria for studies in this review were as follows: primary studies, both observational such as cohort, cross-sectional or case–control, or experimental, such as interventional trials; studies that evaluated the use of DBS for CMV screening, confirmed with at least one gold-standard method (urine or saliva); studies whose population consisted of newborns; and studies available in full text.

Study selection was independently performed by two reviewers. Each reviewer independently conducted the research in the databases. Then, each researcher extracted the studies found and used them in the Rayyan platform, where they also independently selected studies based on the defined inclusion criteria. The discrepancies in the selection process were resolved by consensus or by a third reviewer. Data extraction was presented in Table 2 that summarized the data necessary to answer the research question. The sensitivity and specificity of the DBS PCR methods were established based on a comparison between the DBS method used and samples in urine, saliva, and peripheral blood. The review followed PRISMA-DTA guidelines [10]. Search results were managed in Rayyan platform [11] for title and abstract screening, followed by full-text review of potentially eligible studies; for duplicates, only the most recent version was included.

**Table 2:** Data extraction of the systematic review

ID	Study data			DBS PCR analysis					Diagnostic accuracy of DBS			
Author, year	Country and study period	Study design	Total of participants	Alternative sample used for screening	ConfirmatorySample	DBS Method/assay	Total DBS positive (N)	Total confirmed cCMV	Sensitivity (%)	Specificity (%)	PPV (%)	NPV (%)
Barbi M, 2006 [13]	Italy, Mar 2002 – Feb 2003	Transversal	9,032	-	Urine	Conventional PCR	41	16	-	-	39%	-
Boppana SB, 2010 [14]	USA, Mar 2007 – May 2008	Cohort	20,448	Saliva	Saliva	Single-primer	21	17	28,3%	99,9%	80,9%	99,6%
						2-Primer	12	11	34,4%	99,9%	91,7%	99,8%
Wang S, 2017 [15]	China, Mar 2011 – Aug 2013	Cohort	10,933 (3,953 tested for PCR DBS)	Saliva	Saliva	Conventional PCR	14	11	39,3%	99,9%	78,6%	99,6%
Papaevangelou V, 2017 [16]	Greece, 2008–2010	Cohort	2,149	-	Urine and peripheral blood	Modified extraction	10	10	-	-	100%	-
Noorbakhsh S, 2020 [17]	Iran, July – Aug 2017	Transversal	1,174	-	Urine	Nested PCR on DBS (gB gene)	4	4	-	-	100%	-
Schleiss, 2025 [18]	USA, Feb 2016 – Dec 2022	Cohort	23,641	Urine	Urine	UMN	64	63	72,4%	100%	98,4%	99,9%
						CDC	71	69	79,3%	100%	97,2%	99,9%
Dunn, 2025 [19]	Canada, July 2019 – July 2023	Cohort	551,034	-	Urine	Real-time PCR	689	601	-	-	87,2%	-
Kaye, 2024 [20]	USA, Feb 2023 – Feb 2024	Cohort	60,115	-	Urine	Qualitative real-time PCR	184	176	-	-	95,6%	-
Tavakoli, 2026 [21]	USA, Oct 2023 – Sep 2024	Cohort	208,099	-	Urine	Quantitative PCR	349	273	-	-	78,2%	-

The certainty of evidence for sensitivity and specificity was assessed using the GRADE-DTA approach. Individual studies were evaluated with the QUADAS-2 tool, which examines risk of bias and applicability across patient selection, index test, reference standard, and flow and timing. Risk of bias was classified as high (⊕), low (◯), or unclear (?) [12].

## Results

The initial search yielded 365 articles across the following databases, using the descriptors presented in Table 1: a total of 85 in PubMed (via MEDLINE), 107 in Scopus, 59 in Bireme, 106 in Embase, 5 in LILACS, 0 in SciELO, and 3 in the Cochrane Central Register of Controlled Trials (CENTRAL) (via the Cochrane Library). After removing 210 duplicates, 155 studies remained for title and abstract screening.

Of the 155 remaining studies, 125 were excluded for not meeting the eligibility criteria, for the following reasons: inadequate study design (*n* = 45), lack of evaluation of cytomegalovirus screening (*n* = 30), population not composed of neonates (*n* = 15), evaluation of the test as a diagnostic tool instead of screening (*n* = 10), population restricted to risk groups (*n* = 8), use of samples other than dried blood spots for screening (*n* = 8), DBS not being the primary screening method (*n* = 8), and use of serology instead of PCR (*n* = 3).

Thus, 30 articles were pre-selected for full-text evaluation. Of these, 4 were excluded for not being full published articles, 4 due to missing test or outcome data, 4 due to the absence of a standard reference confirmatory method, 3 because DBS was not the primary screening method, 2 due to the use of DBS as a method for identifying and confirming cases, 2 because the selected population was high-risk, 1 because the population was not composed of neonates, and 1 because of an overlapping population. At the end of the process, 9 studies met all the eligibility criteria defined by the PIRT question and were included in the systematic review, as illustrated in the flowchart (Flowchart 1).

**Flowchart 1:**
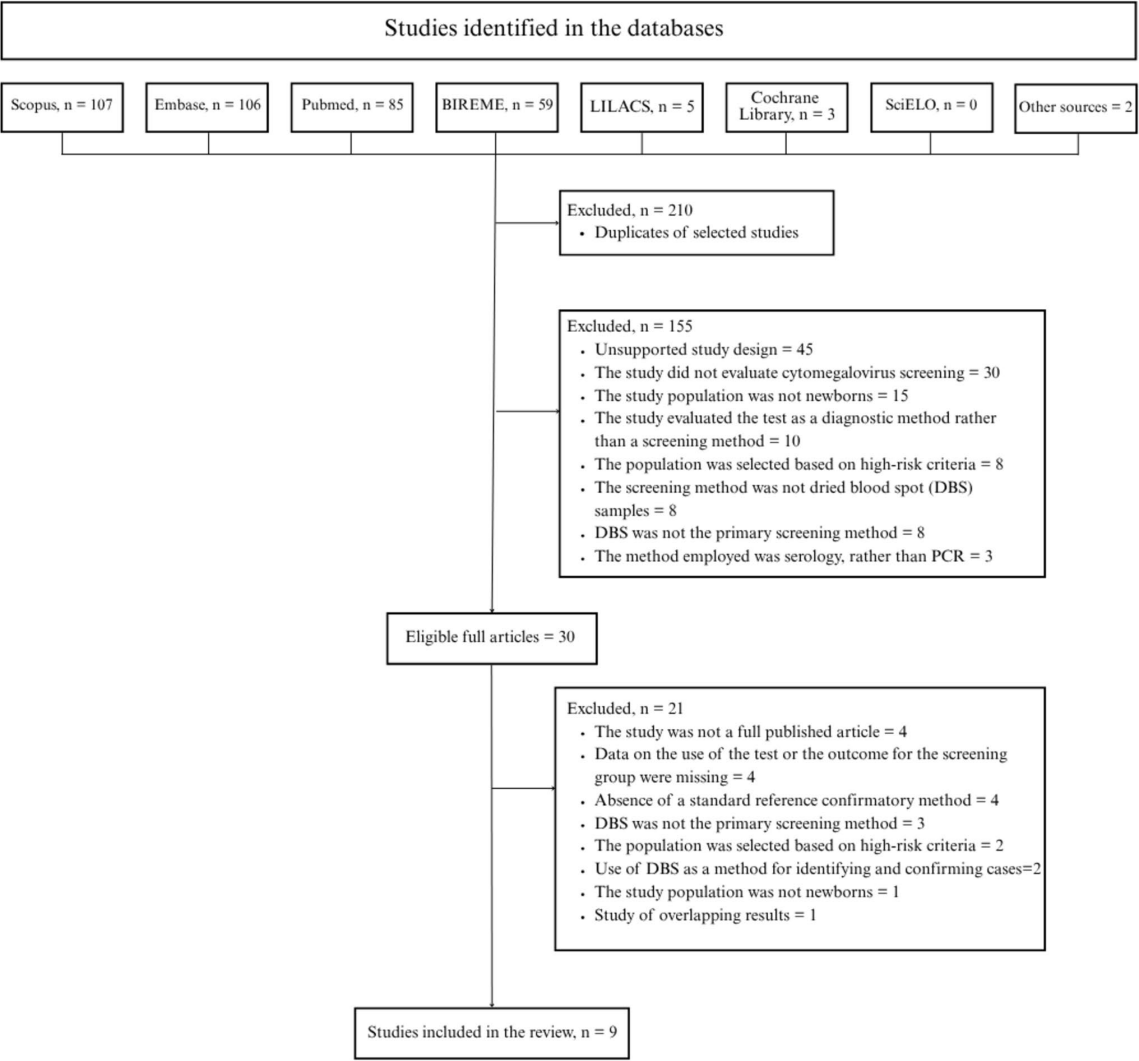
Studies included in the systematic review

Of the 9 selected studies, 7 were cohorts, and 2 were cross-sectional studies. In terms of geographical distribution, 4 studies were conducted in the USA and 1 in each of the following countries: Italy, Canada, China, Iran, and Greece. The included studies tested between 1174 and 551,034 DBS samples each.

Regarding confirmation of DBS screening results, the studies employed the following methods for comparison: seven studies used PCR on urine and two used PCR on saliva. Despite the confirmation that all positive results were performed in urine or saliva samples, only 3 studies used paired samples for cCMV screening (Table 2).

Table 2 below summarizes the studies included in this review, along with their characteristics and diagnostic performance of DBS PCR for screening for congenital CMV infection. The studies were conducted across different countries and time periods. It is noted that most studies modified the cohort design, with heterogeneous sample sizes, ranging from smaller-scale investigations to larger population programs, varying from 1174 to over 550,000 newborns. Urine was the most frequently used confirmatory sample, while saliva was employed both as an alternative screening method and for confirmation in some studies. Only 3 studies reported sensitivity, specificity and NPV. Specificity was often close to or equal to 100%. Sensitivity ranged 28% to over 79%. PPV varied from 39% to values close to 100%. It is important to note that the low PPV (39%) reported by Barbi et al. [13] may be explained by the use of pooled DBS screening, in which samples from 3 newborns were initially tested on a pool and, if positive, they were retested individually.

The PCR techniques used in included studies that provided this information are presented in Table 3.

**Table 3:** The PCR techniques used in included studies for CMV testing in newborns

Author, year	PCR assay	Positive results
Barbi M, 2006 [13]	DNA was extracted from Guthrie cards by thermal shock, and the nested amplification reaction was done in “monotest tubes” (CMV Early Oligo Mix; Amplimedical Bioline, Torino, Italy), by adding the sample DNA and Taq polymerase (Dynazyme II DNA polymerase; Finnzymes, Espoo, Finland) to the reaction mixtures. After the amplification cycles and electrophoretic separation, they detected the 110-bp amplified product. The lower limit of detection of this assay is 400 DNA copies/mL	Each pool was ran in triplicate. If at least 1 of the 3 tests was positive, they were repeted in in triplicate. If any of DBS result was positive confirmation was performed in in a urine sample. If the urine sample was positive, the child entered the clinical and audiologic follow-up schedule.
Boppana SB, 2010 [14]	ABI 7500 system (Applied Biosystems Inc., Foster City, CA). Two distinct assays were used: initially, a single-primer assay targeting the glycoprotein B (gB) region, followed by a two-primer assay that included a second target in the immediate early 2 (IE-2) gene region as an effort to improve sensitivity.	Each sample was analyzed in duplicate, and PCR. If one or more genome copies in both PCR runs were positive, PCR was repeated if salivary detection of early antigen fluorescent foci (DEAFF) who had a negative result in the first PCR run.
Wang S, 2017 [15]	TaqMan-based primers and probes targeting the cytomegalovirus glycoprotein B (gB) gene, in qPCR Mx3000P systems	CMV-positive specimens were retested and ≥ 5 copies of CMV DNA per reaction for saliva or ≥ 1 copy of CMV DNA for DBS samples were considered positive
Noorbakhsh S, 2020 [17]	A nested PCR assay was developed using primers specific for a conserved region of the CMV gB gene. Initially, 7 μl of template DNA were amplified in a 25 μl reaction containing PCR Master Mix, external primers, and sterile water, under the following conditions: initial denaturation at 94 °C for 2 minutes, followed by 35 cycles of 94 °C for 30 s, 58 °C for 45 s, and 72 °C for 60 s, with a final extension at 72 °C for 5 minutes. This procedure generated a 450 bp fragment. In the second round of amplification, using 1 μl of the primary product as template and internal primers with an annealing temperature adjusted to 55 °C, a 220 bp fragment was obtained. The final products were separated by electrophoresis on a 2% agarose gel and visualized under UV light.	DBS samples with positive results in at least 2 of 3 DBS samples were considered as CMV positive and cCMV infection in newborns was confirmed through positive urine PCR tests.
Dunn, 2025 [19]	DNA was extracted from two 3.2-mm DBS punches using a standard Tris hydrochloride extraction method. Initial polymerase chain reaction (PCR) analysis was conducted via a custom test developed in their laboratory to detect the CMV genes UL55 and UL83.	- Samples with any level of CMV detection, or a ribonuclease P (RNaseP) cycle threshold at or above 30 (indicating insufficient DNA in the reaction), were automatically retested- Samples with clear amplification of both UL55 and UL83 were considered positive- Those with weak or discordant amplification were adjudicated and reported as positive or borderline positive or testing was repeated.
Tavakoli, 2026 [21]	Nucleic acid was extracted from two 3.2-mm DBS punches using a commercially available DNA extraction reagent (Extracta DBS; Quantabio). Nucleic acid amplification and detection was achieved using a cCMV PCR reagent kit (NeoMDx; Revvity).	Specimens with an RPP30 PCR result that was out of range [Cycle threshold (Ct) ≥ 30] were re-extracted and reamplified. Specimens with an acceptable RPP30 PCR result (Ct<30) and no CMV amplification (Ct>40) were reported as screen negative for CMV. Specimens with a positive CMV qPCR result (Ct ≤ 40) were re-extracted in duplicate and qPCR was performed in duplicate on each extract. In addition, qPCR was again performed on the original extract. If ≥3 qPCR results from the 6 total PCR results were positive, the neonate was referred for cCMV evaluation.

Meta-analysis was not performed due to substantial methodological and clinical heterogeneity among the included studies. Sources of heterogeneity included variability in PCR techniques, differences in DNA extraction protocols, use of distinct reference standards (saliva versus urine PCR), and the presence of verification bias in several studies, where only DBS-positive cases underwent confirmatory testing. In addition, not all studies provided paired data for sensitivity and specificity calculations. Given these inconsistencies and the lack of comparable outcome measures across studies, quantitative pooling was considered inappropriate.

The quality analysis of each study is presented in Table 4. According to GRADE quality of evidence of the systematic review, most included studies demonstrated a low risk of bias across the domains of patient selection, index test, and reference standard. In large population-based screening studies [13, 16, 17, 19–21], only infants with a positive DBS screening test systematically receive the diagnostic reference standard (urine/saliva PCR within 21 days). Once screened-negative infants were not routinely tested with the reference standard, false-negatives are missed, precluding the direct calculation of sensitivity and specificity. According to the QUADAS-2 framework, this differential verification warrants a high risk of bias classification in the flow and timing domain. One study showed unclear risk in the reference standard domain, reflecting insufficient methodological information. Regarding applicability concerns, most of the studies were considered low concern across all domains, although two of seven studies presented potential limitations in patient selection. The presence of verification bias should be considered when interpreting the diagnostic accuracy of DBS for cCMV screening [12].

**Table 4:** GRADE quality of evidence of the systematic review

Study	Risk of bias				Applicability concerns		
	Patient selection	Index test	Reference standard	Flow and timing	Patient selection	Index test	Reference standard
Barbi M, 2006 [13]	◯	◯	◯	⊕	◯	◯	◯
Boppana SB, 2010 [14]	◯	◯	◯	⊕	◯	◯	◯
Wang S, 2017 [15]	◯	◯	?	◯	◯	◯	◯
Papaevangelou, 2017 [16]	⊕	◯	◯	⊕	⊕	◯	◯
Noorbakhsh, 2020 [17]	⊕	◯	◯	⊕	⊕	◯	◯
Schleiss, 2025 [18]	◯	◯	◯	⊕	◯	◯	◯
Dunn, 2025 [19]	◯	◯	◯	⊕	◯	◯	◯
Kaye, 2024 [20]	◯	?	◯	⊕	◯	⊖	◯
Tavakoli, 2026 [21]	◯	◯	◯	⊕	◯	◯	◯

◯ low risk, ⊖ moderate risk, ⊕ high Risk,? unclear risk

## Discussion

Although all positive results were confirmed using urine or saliva samples, only three out of the nine studies conducted PCR testing on paired samples in every case, from which it was possible to determine the sensitivity and specificity of the DBS-PCR tests. The main findings were that sensitivity ranged from 28.3% to 79.3%, specificity from 99.9% to 100%, positive predictive value (PPV) from 39 to 100%, and negative predictive value (NPV) from 99.6% to 99.9%. Based on the analysis of test sensitivity in relation to the period in which each study was conducted, it is important to note that sensitivity appears to increase over time. For example, the sensitivity reported by Boppana SB [14] (28.3%–34.4%) is considerably lower than that observed by Schleiss [18] (72.4%–79.3%). Furthermore, there is an apparently lack of sensitivity when DBS was rolled out as public health programs: studies with larger populations, such as Dunn [19], Kaye [20], and Tavakoli [21], showed a lower prevalence than expected.

PCR testing in urine within the first 3 weeks of life is considered the gold standard, for cCMV diagnosis [22]. According to Centers for Disease Control and Prevention (CDC) [23], for public health surveillance purposes, confirmatory laboratory evidence criteria include detection of CMV DNA from urine, whole blood (including DBS) or cerebrospinal fluid; however, a negative urine result in a sample collected within 21 days of life rules out cCMV. DBS screening offers important advantages, including feasibility and compatibility with existing screening infrastructure. Nevertheless, the feasibility of implementation of newborn cCMV screening using DBS may vary depending on public health funding and laboratory capacity for newborn screening programs. The number of positive DBS cases and confirmed cCMV cases varies considerably, with larger studies reporting higher absolute numbers but similar prevalences. When available, specificity was consistently high, while sensitivity showed great variability, depending on the method and assay used. PPV also varied widely, reflecting methodological, population, and confirmation strategy differences [24]. It is important to notice that the Barbi et al. [13] study employed screening on a pool of specimens, which may explain the lower PPV compared to other studies.

To clarify the found variation, especially regarding sensitivity, it is important to discuss each factor that may have influenced the results of each study, as well as methodological limitations, PCR assay used and technical factors, patient selection bias, and verification bias.

Across the included studies, differences in DBS sensitivity were largely driven by methodological variability, particularly in DNA extraction and amplification protocols, primer selection, and assay design [13–21]. Variability in sensitivity is largely attributable to differences in DNA extraction and PCR protocols [5, 25–28]. Guidelines also emphasize the difference accuracy of these methods [3, 6], as highlighted in previous systematic reviews [28]. The study by Boppana et al. [14], which evaluated the interim results of the CHIMES study, exemplifies this heterogeneity, with positive rates ranging from 0.13% to 0.18% depending on the number of primers and the purification system used. Similarly, in Wang et al. [15], differences in the populations analyzed and between methods (DBS collected in only part of the cohort and saliva collected from all participants) limit direct comparability between diagnostic strategies. More recently, the study by Schleiss et al. [18] showed that DBS has lower sensitivity compared to saliva, although it maintains high specificity. Sensitivity ranged from 72.4% to 79.3% across different laboratories (UMN and CDC), emphasizing the influence of methodological heterogeneity. Nevertheless, despite technical limitations and although reported prevalence rates were lower than expected, the present review demonstrates the feasibility of implementing DBS in large-scale public health newborn screening programs when resources are available. Overall, these findings raise important unresolved questions regarding which infected infants are most likely to be missed and whether current DBS-based strategies achieve sufficient sensitivity for effective neonatal screening, or if further optimization is required.

Studies evaluating DBS-based cCMV screening within public health programs and pilot population-based initiatives reported generally high positive predictive values, supporting the feasibility of large-scale implementation. Dunn et al. [19] reported a PPV of 87.2%, similarly, Kaye et al. [20] and Takavoli et al. [21] presented a PPV of 95.6% and 78.2%, respectively. However, the lower prevalence observed in these studies, compared with expected cCMV prevalence estimates [19], raises concerns that a proportion of infected newborns may not be detected, potentially reflecting the limited sensitivity of DBS under routine screening conditions. These observations highlighted the need for further research to determine which infants are most likely to be missed and whether optimization of DBS-based methodologies could improve case ascertainment in large-scale screening programs.

Despite its lower sensitivity, the method was able to identify a large proportion of confirmed cases, particularly those with greater clinical relevance. In this context, the distinction between CMV infection and CMV disease is noteworthy. In the study by Schleiss et al. [18], although 87 newborns had confirmed infection, only 21 (24.1%) developed disease, indicating that most cases are asymptomatic. The sensitivity of DBS was higher in detecting symptomatic cases compared to asymptomatic ones, suggesting greater sensitivity in individuals with higher viral loads and increased risk of complications.

Similar findings were reported by Dunn et al. [19] in a large-scale population screening study involving more than 550,000 newborns. Among cases with positive DBS results and diagnostic confirmation, most were confirmed, and the test showed greater ability to detect individuals with higher viral loads. As previously discussed, subsequent confirmatory tests were limited to those infants who screened positive. However, although sensitivity was not assessed, the research noted 19 additional symptomatic cases that had negative DBS screening results. Additionally, the detection rate of 0.13% was lower than observed prevalence reported in other high-income countries, suggesting lower sensitivity, although it must be noted that cCMV prevalence data for Canada are limited.

Nonetheless, although DBS sensitivity remains lower than that of saliva or urine PCR, the current gold-standard methods [22, 29], DBS represents a promising option for large-scale screening. It was shown that DBS PCR has an approximate 90% sensitivity in detection of clinically actionable cCMV cases, depending upon the PCR assay used [18]. As discussed previously, improved methodologies increased sensitivity, enhancing DBS PCR potential of screening. Recently, universal cCMV screening in Canada, Minnesota, and Connecticut was implemented based on DBS PCR test for CMV DNA detection. In Ontario, of the 551.034 newborns who had DBS CMV PCR screening test, 689 screened positive (0.13%) and 601 (87%) had confirmed cCMV infection. Therefore, considering an existing necessary infrastructure in health departments for DBS CMV detection, this method is likely to persist as the selected for universal screening in the future [29].

The lower sensitivity of DBS can also be explained by lower viral loads in neonatal peripheral blood, especially in asymptomatic infections. Saliva and urine contain higher viral loads during the first weeks of life, as CMV replicates more actively in salivary glands and urinary epithelium, accounting for the higher sensitivity of saliva- and urine-based assays [2, 5]. Symptomatic neonates, who typically have higher and prolonged viremia, are more readily detected by DBS [7]. Nevertheless, several studies included in this review reported missed cases even among symptomatic infants, indicating that factors beyond viral load, such as limited blood volume collected on filter paper and variability in PCR methodologies, may also affect DBS diagnostic performance.

Despite discussed limitations, DBS is already incorporated into routine neonatal screening in many countries, representing a practical and scalable approach, particularly where saliva or urine collection is logistically challenging [15]. Evidence suggests that targeted cCMV testing following newborn hearing screening may improve the early identification of infants with cCMV-related hearing loss. In the CHIMES study, newborn hearing screening identified only 57% of infants with cCMV-related sensorineural hearing loss present during the neonatal period, whereas 43% passed the hearing screen despite having confirmed hearing loss. In addition, infants with cCMV who pass newborn hearing screening remain at risk for progressive or late-onset hearing loss. Regardless of these advances, screening strategies for cCMV remain heterogeneous across countries and guidelines. In addition to universal DBS screening, alternative approaches include targeted screening of neonates with risk factors, testing infants who fail hearing screening using urine PCR within the first 3 weeks of life, and maternal serology during pregnancy to detect seroconversion [3, 6]

A major strength of this study is the comprehensive search strategy, which reduces bias and enhances the reliability of the findings by providing a robust synthesis of the available literature [10, 12]. However, this review is limited by the small number of available studies evaluating DBS for neonatal cCMV screening. This scarcity likely reflects ongoing debate regarding DBS sensitivity, particularly in comparison with saliva PCR [7]. Screening studies may have limitations in feasibility due to consent, costs, and sample size, in addition to considering the low prevalence of congenital CMV in the general population. On the other hand, the studies by Dunn et al. [19] and Tavakoli et al. [21] report much larger cohorts, resulting from the implementation of cCMV test as part of the neonatal screening program, which favors the evaluation of this test as a screening tool. In addition, heterogeneity among primary studies, stemming from different comparison samples (saliva, urine, or venous blood), limits direct comparability and generalizability [15]. The lack of standardized laboratory protocols further affects diagnostic performance [25].

In conclusion, neonatal screening for congenital cytomegalovirus using DBS samples is promising, particularly in resource-limited settings, due to its feasibility, low cost, and integration into existing programs, offering high specificity and practical applicability. Despite variable sensitivity, which remains inferior to gold-standard methods, the test shows high specificity and predictive values, supporting its reliable identification of positive cases. Furthermore, laboratory advances may mitigate this limitation. This systematic review reinforces DBS public health importance as a complementary tool for early cCMV diagnosis, in the absence of a licensed CMV vaccine, early diagnosis remains the primary strategy to reduce the burden of congenital infection, enabling interventions that may reduce neurological and hearing sequelae, and support incorporation into public health guidelines. Future efforts should focus on protocol standardization, technological optimization, and strengthened public policies to support the integration of DBS into universal neonatal screening programs.

## Data Availability

No datasets were generated or analysed during the current study.

## Abbreviations

cCMV: Congenital human cytomegalovirus
CDC: Centers for Disease Control and Prevention
CMV: Cytomegalovirus
DBS: Dried blood spots
NPV: Negative predictive value
PCR: Polymerase chain reaction
PPV: Positive predictive value
SNHL: Sensorineural hearing loss
UMN: University of Minnesota
